# Effects of Gibberellin (GA_4+7_) in Grain Filling, Hormonal Behavior, and Antioxidants in High-Density Maize (*Zea mays* L.)

**DOI:** 10.3390/plants9080978

**Published:** 2020-07-31

**Authors:** Wenwen Cui, Quanhao Song, Bingyun Zuo, Qingfang Han, Zhikuan Jia

**Affiliations:** 1 Key Laboratory of Crop Physic-Ecology and Tillage Science in Northwest Loess Plateau, Ministry of Agriculture/College of Agronomy, Northwest A&F University, Yangling 712100, China; zuoby11@163.com (B.Z.); hanqf88@nwafu.edu.cn (Q.H.); 2 Key Laboratory of Agricultural Soil and Water Engineering in Arid and Semiarid Areas, Ministry of Education/Institute of Water-Saving Agriculture in Arid of China, Northwest A&F University, Yangling 712100, China; 3 Zhumadian Academy of Industry Innovation and Development, Huanghuai University, Zhumadian 463000, China; 4 Zhumadian Academy of Agricultural Sciences, Zhumadian 463000, China; songmanl.2005@163.com

**Keywords:** gibberellins (GA_4+7_), grain filling, hormone, antioxidants, yield, maize

## Abstract

Dense plant cultivation is an efficient approach to improve maize production by maximizing the utilization of energy and nutrients. However, dense plant populations may aggravate the abortion rate of young grains, resulting in fewer kernels per ear. The rate and duration of grain-filling play decisive roles in maize grain yield. Therefore, to increase plant density, enhancing the grain-filling rate, extending the growth period of individual maize plants and regulating crop senescence would be the first priority. In this study, we examined the regulatory effects of GA_4+7_ under two application methods: shanks and silks were moistened by cotton full with GA_4+7_ solution at concentrations of 0, 10, 60, and 120 mg L^−1^. The results showed that GA_4+7_ improved the grain-filling rate by increasing the content of auxin, gibberellin, zeatin, and abscisic acid in grains compared to control plants. In addition, the auxin, gibberellin, and zeatin contents in the grains were positively and significantly correlated with the maximum grain weight and the maximum and mean grain-filling rates. Moreover, GA_4+7_ increased the activities of superoxide dismutases, catalases, and peroxidases and reduced the malondialdehyde content in leaves compared with untreated plants. At the concentration of 60 mg L^−1^, GA_4+7_ showed the greatest effect on shank and silk applications (Sh-60 and Si-60) followed by 10 mg L^−1^ (Sh-10) for shank treatment and 120 mg L^−1^ (Si-120) for silk treatment. Our results suggest that a concentration of 60 mg L^−1^ GA_4+7_ for shank and silk application may be efficiently used for changing the level of hormones in grains and antioxidant enzymes in ear leaves, which may be useful for enhancing grain-filling rate and delaying leaf senescence, resulting in an increase in maize grain yield.

## 1. Introduction

Maize is one of the most important cereal crops and is widely used as a food, fodder, and industrial raw material worldwide [1] Recently, the global production of maize has exceeded that of rice and wheat [2]. Additionally, the global population is projected to further increase by 34% in 2050 (a total of 9.15 billion); thus, an estimated 70% increase in agricultural production is demanded [3]. Achieving the food demands for such a large population is a challenge to food security [4]. Dense plant cultivation has the potential to attain higher crop productivity [5,6,7,8], which leads to a greater leaf area index (LAI) and enables crops to use intercepted solar radiation more efficiently [9,10,11,12]. However, dense plant cultivation could greatly affect the grain-filling process and result in lower maximum and average maize grain-filling rates [13,14]. Although dense plant populations increase the number of spikes per unit area but result in a decline of per-plant growth rates and exacerbate young kernel abortion, the ears per plant and kernels per ear have decreased [15,16,17,18]. Therefore, while adopting high planting density in maize, improving the individual maize grain-filling rate is of great concern in modern crop systems.

Grain filling is the ultimate growth stage of cereal caryopse formation when the final kernel weight is established and thereby contributes greatly to grain productivity [19,20]. Plant hormones have been shown to play a significant role in modifying grain filling progress and other various factors that regulate grain filling progress. Yang et al. have reported that the wheat grain-filling rate is mediated by the balance between abscisic acid and ethylene, and the grain-filling rate increases with an increase in the ratio of ABA to ethylene in grains [21]. The zeatin (Z) and zeatin riboside (ZR) in developing seeds have been shown to temporarily improve endosperm cell division and fertilization during kernel setting [22]. The contents of indole-3-acetic acid (IAA) and abscisic acid (ABA) were higher in superior grains than in inferior grains [23], and the increased ABA and reduced IAA shortened the grain-filling period in the inferior grains [24]. Liu et al. and Ali et al. reported a positive and significant correlation of ABA, IAA, and Z + ZR contents with the maximum grain weight and grain-filling rates [25,26]. Additionally, higher gibberellin (GA) contents were present at early stages of grain filling in rice [27]. Ahmad et al. stated that the uniconazole application could improve significantly the Z+ZR, ABA contents and reduce the GA content in the seeds during the process of seed filling [28]. These studies showed that cereal grain filling is markedly affected by alterations in hormone levels in grains.

In recent decades, plant growth regulators (PGRs), including GAs, have attracted the interest of agriculture scientists and are broadly used in agronomic crops. Gibberellins comprise a large family of hormones that are ubiquitous in higher plants and have long been known as endogenous plant growth regulators, promoting several aspects of plant growth and developmental processes, such as cell division, stem elongation, seed germination, dormancy, leaf expansion, flower and fruit development [29,30]. Gibberellins were first discovered in the 1930s by Teijiro Yabuta and Yusuke Sumiki and were named after the pathogenic fungus *Gibberella fujikuroi* [31]. Subsequently, 136 different types of gibberellin structures have been identified and isolated from different fungi and plant sources [32,33]. Despite a large number of GAs, relatively a few gibberellins, such as GA1, GA3, GA4, and GA7, are believed to have intrinsic biological activity in higher plants [30]. GA3 is one of the most widely used plant growth hormones. With the deepening of research, other GA hormones, especially GA4 and GA7, have attracted increasing attention due to their special effects on plants. Some researchers have suggested that GA_4+7_ could successfully induce fruit set and increase fruit size in cucumber, pear, and apple [34,35,36]. In addition, compared to pollinated fruit, GA_4+7_-treated fruits accumulated more quantities of sucrose and fewer organic acids [35]. Therefore, the application of GA_4+7_ proved to be an important agricultural remedy in horticultural crops, which dramatically increase yield and income. However, the application of GA_4+7_ in cereal crops is limited, and the effect of exogenous GA_4+7_ on the regulation of maize grain filling and its physiological mechanism has not been determined.

Reactive oxygen species (ROS) are continually being produced in plants, which can act as a signal molecule in plants and trigger a series of cellular responses [37,38]. In plants, an increase in the ROS levels exceeding the detoxification levels of plant tissues could be toxic. However, plants have evolved an enzymatic and nonenzymatic antioxidant defense mechanism to effectively scavenge ROS and maintain a proper balance within plants. Enzymatic antioxidants mainly include superoxide dismutase (SOD), peroxidase (POD), ascorbate peroxidase (APX), and catalase (CAT). However, senescence and various environmental stresses could disrupt the balance between ROS generation and detoxification, resulting in lipid peroxidation, chlorophyll degradation, and loss of cell membrane integrity [39]. The research of exogenous GA_4+7_ stress on antioxidants in maize has yet to be determined and unclear.

These previous studies have indicated that the potential of GA_4+7_ to control grain filling of maize is limited, and the possible effects on hormone change and antioxidants have not been investigated in detail. Therefore, the objectives of the present study were to investigate the effects of shank and silk treatments with different concentrations of GA_4+7_ on grain-filling rate, hormonal changes and its relationship with grain filling and possible changes in antioxidants. We provided the first detailed description of the GA_4+7_ application in improving the grain-filling rate of maize and antioxidants with the aim of achieving higher grain yield in high-density maize.

## 2. Materials and Methods

### 2.1. Seed and Reagent Source

The hybrid maize seeds (Zhengdan, 958, a local hybrid) were provided by China National Seeds Group Co., Ltd (Beijing, China). The seed moisture content and germination potential were 13% and 90%, respectively. The test reagent Gibberellin GA_4+7_ [*m* (GA4): *m* (GA7) = 40:60] (BR, purity ≥ 90.0%) was purchased from Shanghai Ryon Biological Technology Co., Ltd., Shanghai, China.

### 2.2. Study Site Description

**Figure 1:**
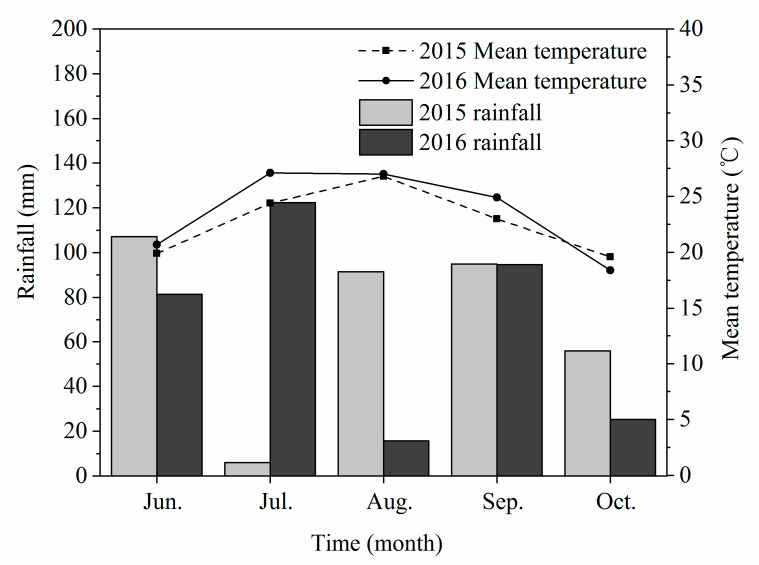
Mean temperature and rainfall during the growing seasons in 2015 and 2016.

Field trials were conducted in 2015 and 2016 at the Institute of Water Saving Agriculture in Semi-Arid Regions of China, Northwest A & F University, Yangling (34°20′ N, 108°04′ E, 454.8 m altitude), Shaanxi Province, China. The climate is a temperate semiarid monsoon with a mean annual temperature of 12–14 °C, and the mean annual precipitation and evaporation were 580.5 mm and 993.2 mm, respectively. The total yearly sunshine duration was 2158 h, and the no-frost period was 221 days. Most of the rainfall occurred in hot seasons from July to September (Figure 1). The soil of the experimental site consists of Cumuli-Ustic Isohumosols (Chinese Soil Taxonomy), and the soil characteristics from 0 to 40 cm in depth at the research site are shown in Table 1.

### 2.3. Experimental Design and Treatments

The regulatory effect of GA_4+7_ under two application methods was studied in 2015 and 2016. In the experiment, shanks and silks were moistened by the cotton that full with GA_4+7_ solution at concentrations 0, 10, 60, and 120 mg L^−1^ at one week after the silking stage (R1), at the rate of 45 mL m^−2^. Sh-0, Sh-10, Sh-60, and Sh-120 represent shank treatments with GA_4+7_ at the rates of 0, 10, 60, and 120 mg L^−1^, Si-0, Si-10, Si-60 and Si-120 represent silk treatments with GA_4+7_ at the rates of 0, 10, 60, and 120 mg L^−1^.

The experiment was laid out in a randomized complete block design (RCBD) using three replications. The sub plot area was 45 m^2^ (5 m × 9 m). Maize seeds were manually sown at a density of 97,500 plants ha^−1^ (plant-plant and row-row distances were 17 and 60 cm) on 13 June and 25 June of 2015 and 2016, and harvested on 14 October and 21 October of 2015 and 2016, respectively. All plots were supplied with 225 kg N ha^−1^ and 120 kg P_2_O_5_ ha^−1^. All P fertilizers and 60% of the N fertilizer were applied at presowing. The remaining 40% of urea (N 46%) was applied as a top dressing at the twelfth leaf stage (V12). During the entire growth period, disease, pest, and weed in each treatment were well-controlled. Irrigation was applied when necessary.

### 2.4. Sampling and Measurements

At the tasseling stage (VT), healthy and uniform maize plants were marked in each plot. Three marked ear leaves from each plot were sampled at 10-day intervals from 15 DAS (days after silking) to maturity, the sampled leaves were stored in liquid nitrogen for the determination of ROS. Three marked ears from each plot were sampled at 7-day intervals from the 14 DAS to maturity, and all grains were sampled from each ear. Each ear was divided into three equal parts on the basis of the length, and the grains growth was removed from each part. The grains were divided into superior, middle, and inferior grains as described by Liu et al. [40]. Half of the seeds were instantly frozen in liquid nitrogen and stored at −80 °C for the hormone measurements, and remaining grains were oven-dried at 75 °C until they reached a constant weight for grain filling characteristics and hundred kernel weight measurements.

#### 2.4.1. Grain-Filling Process

The grain-filling data were fitted using the Richards growth equation [41]:

(1) $$W=\frac{A}{{\left(1+Be^{-kt}\right)}^{1/N}}$$

The grain-filling rate (G) was calculated as the derivative of Equation (1):

(2) $$G=\frac{AkBe^{-kt}}{{\left(1+Be^{-kt}\right)}^{\left(N+1\right)/N}}$$

where W is the grain weight (mg), A is the final grain weight (mg), *t* is the time after anthesis (d), and B, k, and N are coefficients determined by regression analysis. (The grain weight and the final grain weight were the average of the superior, middle, and inferior grains)

#### 2.4.2. Endogenous Hormone

The extraction, purification, and quantification of IAA, ZR, GA, and ABA by an indirect ELISA technique were essentially identical to those described by [25,27,28]. The antigens and monoclonal antibodies against IAA, ZR, GA, ABA, and immunoglobulin G-horseradish peroxidase (IgG-HRP) used in the ELISA were produced at the Phytohormones Research Institute, China Agricultural University, Beijing, China.

For the extraction and purification of IAA, ZR, GA (GA1+GA3), and ABA, a sample of approximately 0.5 g was ground in an ice bath with 4 mL 80% (*v*/*v*) methanol extraction buffer containing 1 mmol L^−1^ butylated hydroxytoluene (BHT) as an antioxidant. The homogenates were incubated at 4 °C for 4 h and centrifuged at the same temperature. Afterwards, the supernatants were taken into a new centrifuge tube, precipitated with 1 mL methanol extraction buffer, incubated for 1 h and centrifuged again. The supernatants were passed through Chromosep C18 columns (C18 Sep-Park Cartridge, Waters Corp, Ma, USA) and prewashed with 5 mL 100% ether and then 5 mL 100% methanol. The hormone fractions were dried with N_2_ and dissolved in 1 mL phosphate-buffered saline (PBS) containing 0.1% (*v*/*v*) Tween-20 and 0.1% (*w/v*) gelatin (pH 7.5) for evaluation by enzyme-linked immunosorbent assay (ELISA).

The quantification of IAA, ZR, GA (GA1+GA3) and ABA was performed using a 50-µL sample (standard sample or the test sample), and 50 µL of the diluted antibody was added to each well of the ELISA plate, and the plate was placed in the wet box at 37 °C for 0.5 h. The plate was then washed 4 times with phosphate-buffered saline (PBS) containing 0.1% (*v*/*v*) Tween-20; next, 100 µL of the diluted IgG-HRP was added to each well, and the plate was placed in the wet box at 37 °C for 0.5 h. Afterwards, the plate was washed 4 times again, and then the color reaction was performed. Specifically, to each well, 100 µL of coloration liquid containing 0.1% (*w/v*) o-phenylenediamine (OPD) and 0.04% (*v*/*v*) H_2_O_2_ in substrate buffer (the substrate buffer contains 5.10 g C_6_H_8_O_7_·H_2_O, 18.43 g Na_2_HPO_4_·12H_2_O and 1 mL Tween-20 per liter with pH 5) was added. The chromogenic reaction was also carried out in the wet box, and the reaction was terminated by adding 50 µL 2 mol L^−1^ H_2_SO_4_. The absorbance of the solution was read at 490 nm by a spectrophotometer.

The logit curve was used to calculate the ELISA results. Hormone concentration was expressed as ng g^–1^ fresh weight.

#### 2.4.3. Antioxidant Enzyme

For enzyme extraction, 0.5 g of fresh leaf samples were ground with 5 mL precooled 50 mM phosphate buffer (pH 7.5). The homogenates were centrifuged at 13,000 rpm for 30 min at 4 °C, and the resulting supernatants were used for the enzyme assay. All enzyme activity data were expressed as U mg^−1^ fresh weight (FW).

Superoxide dismutase (SOD) activity was determined by recording the decrease in the absorbance of nitro-blue tetrazolium (NBT) by the enzyme [42]. The reaction mixture consisted of 1.5 mL 50 mM phosphate buffer (pH 7.8), 0.3 mL 130 mmol L^−1^ methionine, 0.3 mL 750 mol L^−1^ NBT, 0.3 mL 100 mol L^−1^ EDTA-Na_2_, 0.3 mL 20 mol L^−1^ FD and 0.3 mL distilled water and was mixed with 20 μL crude enzyme extract. Control and enzyme solutions were placed at 4000 lux light for 30 min. The blank sample was placed in the dark. The absorbance of the solution was monitored at 560 nm by an ultraviolet spectrophotometer.

Peroxidase (POD) activity was assayed according to Ekmekci and Terzioglu [43]. Then, 20 μL of enzyme extract was drawn and mixed with 3 mL of POD reaction solution. The reaction mixture contained 1.5 mL 50 mM sodium phosphate buffer (pH 6.0), 0.5 mL H_2_O_2_ (30%), 0.5 mL 50 mM guaiacol, and 0.5 mL distilled water. The absorbance values were recorded once every 30 s at 470 nm using an ultraviolet spectrophotometer.

Catalase (CAT) activity was assayed by measuring the decomposition of H_2_O_2_ [44]. The reaction mixture contained 1.5 mL 50 mM sodium phosphate buffer (pH 7.0), 0.2 mL crude extract, 1 mL distilled water and 0.3 mL 100 mM H_2_O_2_. The breakdown of H_2_O_2_ was followed by measurement of the absorbance change at 240 nm.

The lipid peroxidation was determined by measuring the content of malondialdehyde (MDA) by following the procedure of Zhang [45]. Leaf samples (0.5 g) were homogenized in 5 mL of 5% trichloroacetic acid (TCA), and the homogenate was centrifuged at 4000 *g* for 10 min at 25 °C. A 2 mL enzyme solution was drawn and mixed with 3 mL of 2-thiobarbituric acid (TBA) in 20% trichloroacetic acid. The mixture was water-bath heated at 100 °C for 20 min and centrifuged for cooling down. The absorbance of the supernatant was determined at 450 nm, 532 nm, and 600 nm.

#### 2.4.4. Yield and Yield Components

To determine ear characteristics and grain yield, thirty representative plants from each replicate were sampled at physiological maturity. Kernel number ear^−1^ and thousand kernel weight (TKW) were measured on 20 representative ears per plot. TKW was determined after drying a thousand grains at 75 °C to a constant weight. Grain yield was determined at 13.0% moisture content.

### 2.5. Statistical Analysis

Analyses of variance (ANOVA) were determined by using SAS 9.2 software (SAS Institute, Cary, NC, USA). The data from each sampling were analyzed separately. A least significance difference test (LSD) was applied to determine the significance between different treatments (*p* < 0.05).

## 3. Results

### 3.1. Effects of GA_4+7_ on Yield and Yield Component

The results of field experiments showed that GA_4+7_ treatments significantly (*p* < 0.05) enhanced the ear characteristics (ear length and diameter, kernels ear^−1^, and 1000 kernel weight) and grain yield of maize compared with control treatments (Table 2). Compared with the control treatment, the Sh-60 and Si-60 treatments had the best effects on the maize yield followed by Sh-10 and Si-120. The shank treatments affected the grain yield mainly by increasing the thousand grain weight of maize; the two-year average thousand grain weights under Sh-10 and Sh-60 treatments were increased by 24.5 g and 36.99 g, respectively, i.e., by 8.5% and 12.8%. While the silk treatments mainly increased kernel number to improve grain yield, the two-year average kernel number under the Si-60 and Si-120 treatments was increased by 77 and 44 kernels per ear, respectively, i.e., 26.9% and 15.5%, respectively. In 2015, the highest concentration of GA_4+7_ in the shank treatments and the lowest concentration of GA_4+7_ in the silk treatments, i.e., Sh-120 and Si-10, had a relatively small impact on increasing yield compared with the control treatment. However, the grain yields of Sh-120 and Si-10 were still improved by a relatively large amount in 2016. The two-year average grain yields under Sh-10, Sh-60, and Sh-120 treatments were increased by 1050.4 kg ha^−1^, 2132.6 kg ha^−1^, 348.8 kg ha^−1^, i.e., 12.1%, 24.6%, and 4.0%, respectively, compared with Sh-0, whereas those under Si-10, Si-60, and Si-120 treatments were greater by 431.9 kg ha^−1^, 2863.4 kg ha^−1^, 1745.6 kg ha^−1^, respectively, i.e., increased by 5.11%, 33.9%, and 20.7% compared with Si-0.

### 3.2. Effects of GA_4+7_ on Grain-Filling Rate

**Figure 2:**
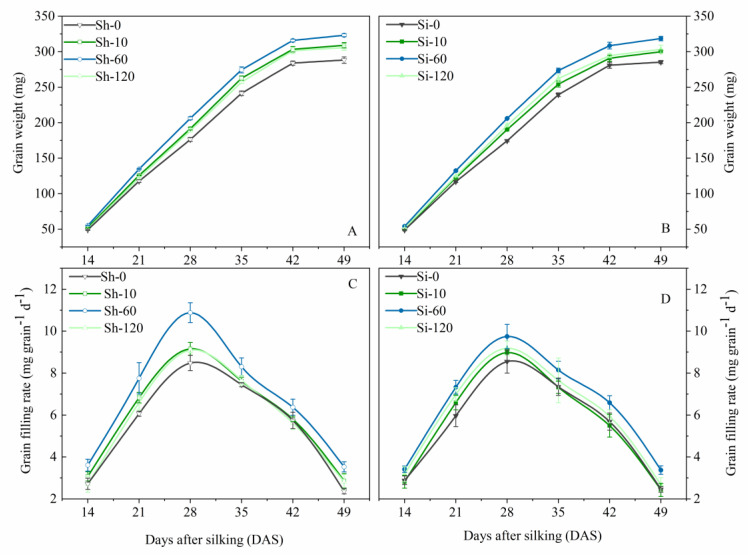
Effects of GA_4+7_ applications on grain weights and grain-filling rates of maize. Sh-0, Sh-10, Sh-60 and Sh-120 represent shank-treatments with GA_4+7_ at the rates of 0, 10, 60, and 120 mg L^−1^, respectively. Si-0, Si-10, Si-60 andSi-120 represent silk-treatments with GA_4+7_ at the rates of 0, 10, 60, and 120 mg L^−1^, respectively. The vertical bars represent the ± the standard error of the mean (n = 3).

GA_4+7_ treatments showed positive effects on grain filling of maize (Figure 2 and Table 3). The grain-filling rate reached was maximized at 28 DAS for all treatments. The maximum grain-filling rate under Sh-10, Sh-60, and Sh-120 treatments was increased by 0.674 mg grain^−1^ d^−1^, 2.399 mg grain^−1^ d^−1^, 0.609 mg grain^−1^ d^−1^, i.e., 7.9%, 28.3%, and 7.2%, respectively, compared with Sh-0, whereas that of Si-10, Si-60 and Si-120 treatments were greater by 0.429 mg grain^−1^ d^−1^, 1.194 mg grain^−1^ d^−1^, 0.633 mg grain^−1^ d^−1^, respectively, i.e., increased by 5.02%, 14.0%, and 7.4% compared with Si-0. GA_4+7_ application at the rate of 60 mg L^−1^ (Si-60 and Sh-60 treatments) evidently improved the grain weights and the maximum and mean grain-filling rates compared to the control and other treatments. Compared with the control, the grain-filling rate in the Sh-60 treatment was increased by 32.7%, 27.8%, 28.3%, 11.5%, 11.1% and 49.0% at 14, 21, 28, 35, 42, and 49 DAS, respectively, while the Si-60 treatment was increased by 18.2%, 22.8%, 14.0%, 10.9%, 15.8%, and 37.4% at the respective growth stages. The grain-filling rate and grain weight of Sh-10 and Si-120 at all stages were only inferior to those of Sh-60 and Si-60. Conversely, Sh-120 and Si-10 had no significant effects on the maximum grain-filling rate but had a significant effect on the mean grain filling and the maximum grain weight compared with control treatments.

### 3.3. Effect of GA_4+7_ on Hormonal Changes in Grains

#### 3.3.1. IAA and ZR Contents in Grains

**Figure 3:**
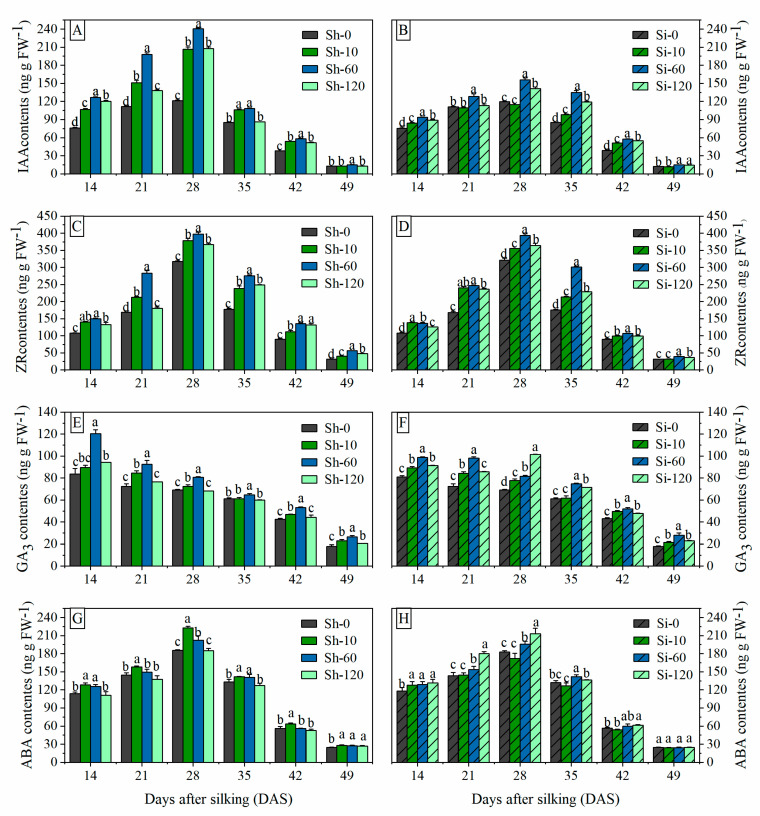
Effects of GA_4+7_ applications on IAA, ZR, GA3 and ABA contents in maize. Sh-0, Sh-10, Sh-60 and Sh-120 represent shank-treatments with GA_4+7_ at the rates of 0, 10, 60, and 120 mg L^−1^, respectively. Si-0, Si-10, Si-60 andSi-120 represent silk-treatments with GA_4+7_ at the rates of 0, 10, 60, and 120 mg L^−1^, respectively. The vertical bars represent the ± the standard error of the mean (n = 3).

The IAA and ZR contents in maize grains exhibited a similar pattern during the grain filling process. The IAA and ZR contents increased linearly during the initial grain-filling stage and attained the maximum peak curves at 28 DAS for all treatments (Figure 3A–D). The IAA and ZR contents under shank-treatments were greater than those under Sh-0 from 14 to 28 DAS. Compared with Sh-0, the average IAA contents from 14 to 28 DAS under Sh-10, Sh-60 and Sh-120 were increased by 50.2%, 82.7%, and 50.7%, respectively, while the average ZR contents from 14 to 28 DAS increased by 23.6%, 41.0%, and 14.6% under Sh-10, Sh-60 and Sh-120 treatments, respectively. The IAA contents in grains under the Si-60 and Si-120 treatments were greater than those under Si-0 from 14 to 49 DAS, respectively. Compared with Si-0, the mean IAA contents at all sampling stages of Si-60 and Si-120 were raised by 32.4% and 20.3%, respectively. At 49 DAS, Si-10 treatments have no significant effects on ZR contents in maize grains; however, the ZR contents in the grains under other shank-smearing and silk-treatments in every sampling period were significantly higher than those under Sh-0 and Si-0. The mean ZR contents under Sh-10, Sh-60, and Sh-120 treatments were increased by 25.5%, 45.2%, and 24.0%, respectively, compared with Sh-0, while those of Si-10, Si-60, and Si-120 increased by 20.7%, 37.0%, and 22.0%, respectively, compared with Si-0.

#### 3.3.2. GA Contents in Grains

The GA contents in the grains exhibited a gradually decreasing trend during grain filling, except for the Si-120 treatment (Figure 3E,F). The Si-120 treatment decreased the level of GA in grains from 14 to 21 DAS, increased abruptly to a peak of 28 DAS and then decreased steadily after reaching its peak. The trend is different from other treatments, and it is necessary to deepen the research to explore the mechanism of its raise. Compared with the control treatments, the Sh-60 and Si-60 treatments significantly increased the GA contents in grains followed by Sh-10 and Si-120. The average GA contents under Sh-10, Sh-60, and Sh-120 treatments during the whole grain filling stage were increased by 9.1%, 26.4%, and 5.1%, respectively, compared with Sh-0, while those of the Si-10, Si-60, and Si-120 treatments were greater by 11.6%, 25.9%, and 22.3%, respectively, compared with Si-0.

#### 3.3.3. ABA Contents in Grains

The ABA content increased rapidly at the early grain-filling stage, reached a maximum at 28 DAS, and then declined progressively in later stages. At the same sampling period, ABA content decreased with increasing GA_4+7_ concentration under shank-treatments, while that under silking-treatments gradually increased with increasing GA_4+7_ concentration (Figure 3G,H). Sh-10 and Si-120 treatments have significant effects on ABA contents in grains at all sampling periods followed by Si-60 and Sh-60. The average ABA contents during the whole grain filling stages under Sh-10 and Sh-60 treatments were increased by 14.0 ng g FW^−1^ and 7.4 ng g FW^−1^, i.e., 12.8% and 7.4% increases, respectively, compared with Sh-0, whereas that of Si-60 and Si-120 increased by 7.9 ng g FW^−1^ and 14.9 ng g FW^−1^, respectively, i.e., 7.9% and 13.6% increases compared with Si-0. However, Sh-120 and Si-10 had no significant effect on increasing ABA content compared with the control treatment (CK).

### 3.4. Effects of GA_4+7_ on Antioxidant Enzymes

#### 3.4.1. SOD Activity

**Figure 4:**
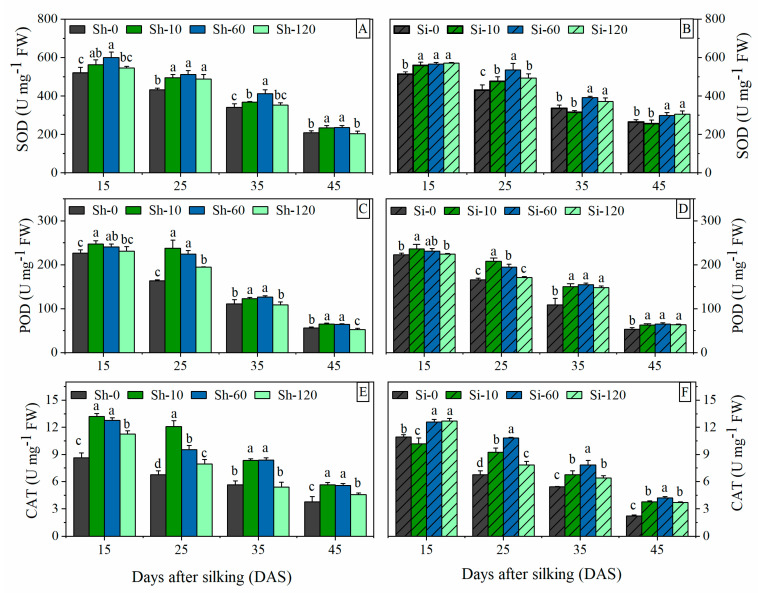
Effects of GA_4+7_ applications on SOD, POD and CAT activities in maize. Sh-0, Sh-10, Sh-60 and Sh-120 represent shank-treatments with GA_4+7_ at the rates of 0, 10, 60, and 120 mg L^−1^, respectively. Si-0, Si-10, Si-60 andSi-120 represent silk-treatments with GA_4+7_ at the rates of 0, 10, 60, and 120 mg L^−1^. The vertical bars represent the ± the standard error of the mean (n = 3).

The SOD activity showed a downward trend from 15 to 45 DAS under all treatments (Figure 4A,B). The SOD activity was significantly improved in GA_4+7_-treated plants at a varied level compared to the control. The 10 mg L^−1^ (Sh-10 and Si-10) and 60 mg L^−1^ (Sh-60 and Si-60) GA_4+7_ treatments had significant effects for improving SOD activity compared with the highest concentration of 120 mg L^−1^ GA_4+7_ (Sh-120 and Si-120 treatments) and the control. The SOD activity in Sh-120 treatment was significantly greater than that in Sh-0 at 25 DAS, and no significant difference was associated with the control at other stages. Si-120 treatment significantly increased SOD activity compared with Si-0 from 15 DAS to 45 DAS. The SOD activity revealed an increase of 12.7%, 17.2%, and 5.9% (mean of all sampling stages) in Sh-10, Sh-60 and Sh-120 treatments compared to Sh-0, while that of Si-10, Si-60 and Si-120 treatments were 12.3%, 16.6%, and 9.3% higher than Si-0.

#### 3.4.2. POD Activity

The POD activity in ear leaves displayed a gradually decreasing pattern of change from 15 to 45 DAS. At the same stage, with the increase of GA_4+7_ concentrations, POD activity decreased gradually (Figure 4C,D). Sh-10 and Sh-60 treatments had the best effect on increasing POD activity. There was no significant difference between Sh-10 and Sh-60. The POD activity revealed a significant increase of 9.0%, 45.6%, 10.8%, and 15.8% in Sh-10-treated plants compared to Sh-0 at 15, 25, 35, and 45 DAS, respectively, while that of Sh-60 was 6.1%, 37.4%, 14.2%, and 15% higher than Sh-0, respectively. In contrast, at the early grain filling stage (from 15 to 25 DAS), the POD activity of Sh-120 is higher than that of Sh-0, then the POD activity of Sh-120 decreased rapidly, and at 45 DAS, the POD activity of Sh-120 was significantly lower than that in Sh-0. The treatments of silk-smearing with GA_4+7_ significantly increased the POD activity at all sampling stages compared with Si-0. At 15 and 25 DAS, Si-10 had the best effects on increasing the POD activity followed by Si-60. From 35 to 45 DAS, the POD activities of Si-10, Si-60, and Si-120 were not significantly different from each other. Si-10, Si-60, and Si-120 increased the average POD activities of the whole grain filling stages by 17.8%, 18.0%, and 15.3%, respectively.

#### 3.4.3. CAT Activity

The application of GA_4+7_ had obvious effects on the activity of CAT and showed a gradually decreasing trend from 15 to 45 DAS (Figure 4E,F). With GA_4+7_ shank-smearing applications, Sh-10 treatment showed the best effect of CAT activity at all sampling stages, followed by Sh-60. At 25 DAS, the CAT activity of Sh-60 was significantly less than that of Sh-10, but there was no significant difference in CAT activity between Sh-10 and Sh-60 in other sampling periods. The CAT activity in Sh-120 was significantly higher than that of Sh-0 and significantly less than that of Sh-60. All concentrations of GA_4+7_ silk-treatments significantly increased CAT activity compared with Si-0. The CAT activity of Si-60 was significantly higher than that of Si-0, Si-10, and Si-120. The average CAT activity of all sampling stages in the Sh-10, Sh-60, and Sh-120 treatments was increased by 58.4%, 46.1%, and 17.7%, respectively, compared with Sh-0. Similarly, the Si-10, Si-60, and Si-120 treatments improved the average CAT activity by 20.2%, 39.8%, and 24.8%, respectively, compared with Si-0.

#### 3.4.4. MDA Contents

**Figure 5:**
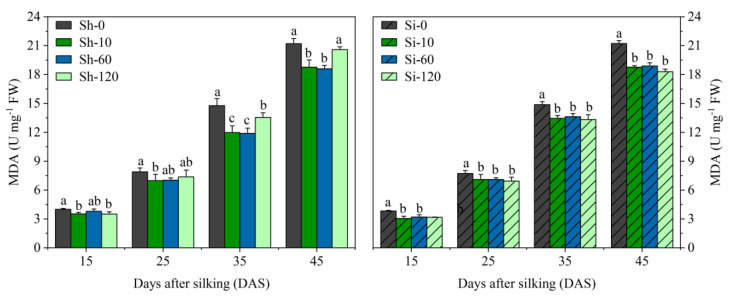
Effects of GA_4+7_ applications on MDA content in maize. Sh-0, Sh-10, Sh-60 and Sh-120 represent shank-treatments with GA_4+7_ at the rates of 0, 10, 60, and 120 mg L^−1^, respectively. Si-0, Si-10, Si-60 andSi-120 represent silk-treatments with GA_4+7_ at the rates of 0, 10, 60, and 120 mg L^−1^, respectively. The vertical bars represent the ± the standard error of the mean (n = 3).

The MDA content increased gradually from 15 to 45 DAS in all the treatments, with the increase in GA_4+7_ treatments being significantly lower than that of the untreated control (Figure 5). All the silking-treatments (Si-10, Si-60, and Si-120) had significant effects on decreasing the MDA from 15 to 45 DAS and were not significantly different from each other. The Sh-10 and Sh-60 treatments significantly reduced the MDA contents at all sampling times and were not significantly different from each other. Moreover, Sh-120 treatment exhibited relatively higher MDA contents than Sh-10 and Sh-60 treatments, but relatively lower MDA contents than Sh-0 at 15 and 35 DAS. The MDA content in Sh-10 treatment was reduced by 11.8%, 11.7%, 19.0%, and 11.5%, while that of Sh-60 treatment was reduced by 11.9%, 10.0%, 19.5%, and 12.3% compared to Sh-0 at 15, 25, 35, and 45 DAS, respectively. The average MDA content from 15 to 45 DAS in the Si-10, Si-60, and Si-120 treatments was reduced by 1.36 U mg^−1^ FW, 1.43 U mg^−1^ FW, 1.22 U mg^−1^ FW, i.e., 11.5%, 12.0%, and 10.2% reduced, respectively, compared with Si-0.

### 3.5. Economic Benefit Analysis of Applying GA_4+7_ in Maize

Table 4 shows the economic benefit analysis of GA_4+7_ smearing application in maize. Net incomes of the treatments at the rate of 10 mg L^−1^ (Sh-10 and Si-10) were not significantly different compared with the control in 2015, but significantly higher than that of the control in 2016. The net income of Sh-60, Si-60, and Si-120 were significantly higher than that of the in 2015 and 2016, respectively. Sh-120 significantly lower net income than that of the control in 2015 and 2016. The average net income in the Sh-10, Sh-60 treatments was increased by 8.85%, 20.19%, while that of Sh-120 was reduce by 12.76%, respectively, compared with Sh-0. Similarly, the Si-10, Si-60, and Si-120 treatments improved the average net income by 5.54%, 39.94%, and 15.1%, respectively, compared with Si-0.

## 4. Discussion

### 4.1. Effects of GA_4+7_ Smearing Application on Grain Yield

The present study illustrates the positive effects of GA_4+7_ on the grain yield of high-density maize by regulating the endogenous hormone levels in grains and the activities of antioxidant enzymes in leaves to promote the grain-filling rate. Previously, GA_4+7_ have been shown to be an effective growth regulator for several crops that could increase the yield and improve the income for farmers [34,35,36]. In our study, GA_4+7_ at a rate of 10 (Sh-10) and 60 (Sh-60) mg L^−1^ under shank-smearing application significantly increased the grain yield of maize in 2015 and 2016. With the silk-smearing application, GA_4+7_ at rate of 60 (Si-60) and 120 (Si-120) mg L^−1^ significantly improved the maize yield in both 2015 and 2016. The application of GA_4+7_ improved the agronomic characteristics of ears, increasing the kernel number per ear and thousand kernel weight. The yield potential of maize can be divided into three key components: kernel number per ear, grain weight, and number of ears per plant. However, the average thousand grain weights under shank-treatments in 2015 and 2016 were higher than that in silk-treatments, and the average kernel number of silk-treatments in 2015 and 2016 were higher than that in shank-treatments. These results indicated that GA_4+7_ with shank-smearing application improved the yield mainly by increasing the grain weight, while silk-smearing with GA_4+7_ increased the kernel number per ear to promote the maize yield.

### 4.2. Relationship of Hormone Changes and Maize Grain Filling

Cytokinins (CTKs) play a significant role in the regulation of the grain filling process, and high CTK levels in maize spike tissues are markedly associated with kernel development [46,47]. CTKs are generally found in the endosperm of developing seeds and may be required for cell division during the early phase of seed setting in other cereal crops [47,48,49,50,51]. In addition to CTKs, IAA also plays a significant role in regulating grain filling [52]. Xu et al. and Fu et al. suggested that regulation of the IAA content in grains could increase the weight of inferior grains [24,53,54]. In the present study, the ZR and IAA contents in the maize grains showed a significantly positive correlation with the maximum grain weight and the maximum and mean grain-filling rates (Table 5). In addition, the changes in ZR and IAA contents in grains during grain filling stages showed a similar pattern. The ZR and IAA contents in the grains transiently increased in the early grain-filling stage and then decreased, reaching a maximum at 28 DAS. We also observed that the IAA and ZR contents between GA_4+7_-treated plants and untreated maize were significantly different at the early filling stage. However, with the development of the grain filling process, the difference in IAA and ZR contents between GA_4+7_-treated maize and control gradually narrowed (Figure 3 and Figure 4). The researchers suggested that auxin and CTKs control endosperm cell growth and division [25,53]. Seth and Singh stated that high IAA levels in a sink organ can create an “attractive power”, leading to increased cytokinin levels in grains [55,56]. These previous findings, as well as the results of this research, indicate that IAA and ZR contents may control early grain filling in maize.

Furthermore, ABA and GAs also play important roles in regulated grain filling. Yang et al. suggested that a higher ABA concentration in grains enhanced the remobilization of prestored carbon to the grains and accelerated the grain-filling rate [27]. Previous studies showed that ABA content in grains was significantly and positively correlated with the maximum and mean grain-filling rates and maximum grain weight [25,26,40]. In our present study, the ABA content in grains was increased by exogenous GA_4+7_, and there was a positive correlation between ABA and grain-filling rate (Table 5). However, the correlation between GAs and grain-filling rate and grain weight were not consistent. Liu et al. and Liu et al. suggested that the content of GAs in the grains was not significantly correlated with the maximum grain weight or the maximum and mean grain-filling rates [25,40]. In contrast, our findings are consistent with the findings of Ali et al. [26], who found that GAs were significantly correlated with the maximum grain weight and the maximum and mean grain-filling rates. Yang et al. stated that spraying exogenous GA reduced the ratio of ABA to GA in the grains [57]. White et al. suggested that GA promote vivipary in maize, while ABA promotes dormancy and GA antagonizes ABA signaling in developing maize embryos, and that the changing hormone balance provides temporal control over the maturation phase [58,59]. These results indicated that the application of exogenous GA_4+7_ changes the content of ABA and GA in the grains, and a hormonal balance, rather than individual hormone content, regulates and improves the maize grain filling.

However, exogenous GA_4+7_ changed the level of endogenous hormones in maize ear and affected the grain filling level of maize, but the role of endogenous hormones in this process as it is unclear if they are a cause or result of the enhanced gain filling in response to GA_4+7_ treatments, we would explore this problem through in-depth study.

### 4.3. Effects of GA_4+7_ Smearing Application on the Antioxidant Enzymes

Reactive oxygen species (ROS) are continually being produced in plants, which can act as a signal molecule in plants and trigger a series of cellular responses [38]. In plants, an increase in the ROS levels exceeding the detoxification levels of plant tissues could be toxic. However, plants have evolved an enzymatic and nonenzymatic antioxidant defense mechanism to effectively scavenge ROS and maintain a proper balance within plants [60]. Enzymatic antioxidants mainly include superoxide dismutase (SOD), peroxidase (POD), ascorbate peroxidase (APX), and catalase (CAT). However, senescence and various environmental stresses could disrupt the balance between ROS generation and detoxification, resulting in lipid peroxidation, chlorophyll degradation and loss of cell membrane integrity [61]. Indeed, our results showed a remarkable increase in the activities of antioxidant enzymes, including SOD, POD, and CAT, and decreased MDA accumulation in the GA_4+7_-treated maize plants at various growth stages. These trends could be interpreted as the enhanced scavenging capacity of reactive oxygen species and reduced membrane lipid oxidation of treated plants. It is well known that membrane lipid peroxidation can cause damage to chloroplast structure and reduce photosynthesis of plants [62]. In this study, GA_4+7_ reduced membrane lipid oxidation of treated plants, therefore, the functional photosynthetic period of leaves was prolonged. Achard et al. showed that GA can degrade DELLA proteins that promote expression of SOD under stress and thereby reduce ROS levels [63]. In our study, GA_4+7_ increased the level of GA, this maybe the reason that GA_4+7_ reduced the ROS in treated-plants.

With the knowledge that developing seeds contain large amounts of hormones, which possess the ability to induce directional movement of nutrients within plants, it appears that leaves senesce when a “sink” (young developing leaves or seeds) needs nutrients to be moved from the rest of the plant. Seth and Wareing suggested that hormone-directed transport plays an important role in directing the movement of nutrients towards developing seeds [55]. These results indicated that the application of exogenous GA_4+7_ changed the level of hormones in seeds, thereby affecting the antioxidant enzymes in leaves and the senescence of maize. However, there is little research about the effect of exogenous GA_4+7_ on reactive oxygen metabolism in maize, and the specific mechanism governing this effect warrants further study.

## 5. Conclusions

Application of GA_4+7_ under high density leads to an increase in the level of IAA, ZR, GA, and ABA. This increase is believed to improve the grain-filling rate, grain weight and number of kernels ear^−1^, as well as increasing the yield. In addition, GA_4+7_ application improves the activities of antioxidant enzymes in leaf. The grain filling period can be prolonged by delaying the senescence of maize such that the grain filling is sufficient and eventually increases the yield. Our results show the positive effect of GA_4+7_ on maize grain-filling rate and antioxidant enzymes and may effectively be used for crop improvement, especially for cereal crops. At the rate of 60 mg L^−1^, GA_4+7_ showed the greatest effect for shank smearing and silk smearing (Sh-60 and Si-60) followed by 10 mg L^−1^ (Sh-10) for shank smearing and 120 mg L^−1^ (Si-120) for silk smearing. The results from the present study illustrate that GA_4+7_ application may be used efficiently for altering the level of hormones in grains and antioxidant enzymes in ear leaves, which may be useful for enhancing the grain-filling rate and increasing the grain yield of maize.

## Figures and Tables

**Table 1:** Soil chemical properties of the top 40 cm in the experimental fields at the Institute of Water Saving Agriculture in Semi-Arid Regions of China in Northwest A&F University, Yangling, Shannxi Province, China, in 2015 and 2016.

Soil Layer (cm)	SOM (g kg^−1^)	STN (g kg^−1^)	STP (g kg^−1^)	STK (g kg^−1^)	SAN (mg kg^−1^)	SAP (mg kg^−1^)	SAK (mg kg^−1^)
0–20	10.91	1.41	0.82	5.86	58.41	26.18	95.98
20–40	8.85	0.99	0.73	4.99	46.30	19.85	68.24

Note. SOM: soil organic matter; STN: soil total nitrogen; STP: soil total phosphorus; STK: soil total potassium; SAN: soil available nitrogen; SAP: soil available phosphorus; SAK: soil available potassium.

**Table 2:** Effects of GA_4+7_ on ear length (cm), ear diameter (mm), kernels ear^−1^, thousand grain weight (g), and grain yield (t hm^−2^) of maize in 2015–2016.

Year	Treatments	Kernel Number (No.ear^−1^)	Ear Length (cm)	Ear Diameter (mm)	Thousand Kernel Weight (g)	Grain Yield (t hm^−2^)
2015	Sh-0	477 ± 7ab	14.1 ± 0.4b	45.6 ± 0.4b	284.1 ± 7.1b	8.3 ± 0.3c
	Sh-10	474 ± 18ab	14.9 ± 0.2a	46.2 ± 1.1b	311.9 ± 9.6a	9.5 ± 0.3b
	Sh-60	501 ± 12a	14.9 ± 0.1a	49.7 ± 0.7a	319.1 ± 4.1a	10.5 ± 0.7a
	Sh-120	470 ± 17b	14 ± 0.5b	45.3 ± 0.9b	294.2 ± 8.8b	8.9 ± 0.5bc
	Si-0	475.1 ± 2.3b	14.1 ± 0.5b	45.8 ± 0.1b	285.1 ± 6.2c	8.2 ± 0.22c
	Si-10	462.1 ± 25.2b	14.1 ± 0.3b	45.3 ± 0.7b	297 ± 2b	8.16 ± 0.49c
	Si-60	523 ± 20.1a	15.4 ± 0.5a	47.1 ± 0.5a	311.7 ± 6.5a	10.88 ± 0.14a
	Si-120	489.2 ± 19.9ab	14.6 ± 0.3ab	46.8 ± 0.3a	305.4 ± 6.2ab	9.89 ± 0.17b
2016	Sh-0	512 ± 2c	15.4 ± 0.2c	46.8 ± 1b	294.1 ± 4.6c	8.8 ± 0.5c
	Sh-10	582 ± 4b	16.3 ± 0.2b	49.5 ± 0.5a	315.3 ± 3.9b	9.9 ± 0.5b
	Sh-60	602 ± 10a	17.3 ± 0.7a	50.2 ± 0.4a	329.8 ± 6.5a	11.1 ± 0.1a
	Sh-120	577 ± 3b	16.7 ± 0.5ab	48 ± 0.9b	312 ± 3.9b	9.1 ± 0.4bc
	Si-0	506 ± 6c	15.2 ± 0.5b	46.9 ± 0.5c	290.8 ± 7.5b	8.7 ± 0.03d
	Si-10	581 ± 6b	17.2 ± 0.1a	49.7 ± 0.2a	310.3 ± 7.1a	9.6 ± 0.02c
	Si-60	613 ± 9a	17.5 ± 0.4a	49.3 ± 0.6a	320.9 ± 3.9a	11.74 ± 0.76a
	Si-120	581 ± 7b	17.4 ± 0.2a	48.3 ± 0.5b	307.7 ± 9.6a	10.5 ± 0.05b

Sh-0, Sh-10, Sh-60 and Sh-120 represent shank-treatments with GA_4+7_ at the rates of 0, 10, 60, and 120 mg L^−1^, respectively. Si-0, Si-10, Si-60 andSi-120 represent silk-treatments with GA_4+7_ at the rates of 0, 10, 60, and 120 mg L^−1^, respectively. Data are expressed as the mean ± S.D. (n = 3). Means followed by different letters within a column are significantly different at *p* < 0.05 as determined by the LSD test.

**Table 3:** Grain-filling characteristics of maize under different GA_4+7_ smearing applications.

Treatments	Wmax (mg)	Gmax (mg Grain^−1^ d^−1^)	Gmean (mg Grain^−1^ d^−1^)
Sh-0	288.3 ± 4.51c	8.48 ± 0.37c	5.47 ± 0.08c
Sh-10	309.0 ± 3.81b	9.16 ± 0.31b	5.89 ± 0.15b
Sh-60	323.2 ± 2.2a	10.88 ± 0.47a	6.74 ± 0.05a
Sh-120	305.8 ± 3.86b	9.09 ± 0.12bc	5.75 ± 0.11b
Si-0	285.3 ± 2.18c	8.55 ± 0.54b	5.48 ± 0.24c
Si-10	300.1 ± 2.09b	8.98 ± 0.14ab	5.61 ± 0.03bc
Si-60	318.5 ± 3.09a	9.74 ± 0.58a	6.43 ± 0.06a
Si-120	303.6 ± 5.64b	9.18 ± 0.35ab	5.93 ± 0.37b

Sh-0, Sh-10, Sh-60 and Sh-120 represent shank-treatments with GA_4+7_ at the rates of 0, 10, 60, and 120 mg L^−1^, respectively. Si-0, Si-10, Si-60 andSi-120 represent silk-treatments with GA_4+7_ at the rates of 0, 10, 60, and 120 mg L^−1^, respectively. Data are expressed as the mean ± S.D. (n = 3). Means followed by different letters within a column are significantly different at *p* < 0.05 as determined by the LSD test. Wmax: the final grain weight; Gmax: maximum grain-filling rates; Gmean: mean grain-filling rates.

**Table 4:** Economic benefit analysis of applying GA_4+7_ in maize.

Years	Treatments	CC (Yuan hm^−2^)	FC (Yuan hm^−2^)	LC (Yuan hm^−2^)	GC (Yuan hm^−2^)	TC (Yuan hm^−2^)	YI (Yuan hm^−2^)	NI (Yuan hm^−2^)
2015	Sh-0	1325	2603	370	0	4298	16,740	12,442b
	Sh-10				126	4424	17,100	12,676b
	Sh-60				756	5054	18,900	13,846a
	Sh-120				1512	5810	16,020	10,210c
	Si-0	1325	2603	370	0	4298	14,760	10,462c
	Si-10				126	4424	14,690	10,266c
	Si-60				756	5054	19,580	14,526a
	Si-120				1512	5810	17,800	11,990b
2016	Sh-0	1325	2603	370	0	4298	16,720	12,422c
	Sh-10				126	4424	18,810	14,386b
	Sh-60				756	5054	21,090	16,036a
	Sh-120				1512	5810	17,290	11,480d
	Si-0	1325	2603	370	0	4298	16530	12,232c
	Si-10				126	4424	18,240	13,816b
	Si-60				756	5054	22,306	17,252a
	Si-120				1512	5810	19,950	14,140b

CC: Cultivation cost, includes the cost of farming and sowing machinery and labor, the cost of seed and pesticide, the cost of field management. FC: Fertilizer cost, the nitrogen fertilizer used is urea (3.9 yuan kg^−1^, nitrogen content N 46%), phosphorus fertilizer is diamine phosphate (4.2 yuan kg^−1^, including N 18%, P2O5 46%).LC:Labour cost of smearing apply. GC: the cost of GA_4+7_ (28yuan g^−1^). TC: total cost. YI: income of yield, the market price of corn in 2015 was about 1.8 yuan kg^−1^, and the market price of corn in 2016 corn was 1.9 yuan kg^−1^. NI: net income. Means followed by different letters within a column are significantly different at *p* < 0.05 as determined by the LSD test.

**Table 5:** Correlation coefficients of mean hormone contents in maize grain with the maximum grain filling rate (Gmax), mean grain filling rate (Gmean), and maximum grain weight (Wmax) of maize.

	Wmax	Gmax	Gmean
IAA	0.889 **	0.866 **	0.832 *
ZR	0.988 **	0.914 **	0.908 **
GA3	0.859 **	0.841 **	0.897 **
ABA	0.481	0.376	0.495

IAA, indole-3-acetic acid; ABA, abscisic acid; ZR, zeatin riboside; GA3, gibberellin 3; Wmax: the final grain weight (mg); Gmax: maximum grain-filling rates; Gmean: mean grain-filling rates. * Significant at the 0.05 probability level (n = 8). ** Significant at the 0.01 probability level (n = 8).
